# *Brachystemma calycinum* D. Don Effectively Reduces the Locomotor Disability in Dogs with Naturally Occurring Osteoarthritis: A Randomized Placebo-Controlled Trial

**DOI:** 10.1155/2012/646191

**Published:** 2012-07-15

**Authors:** Maxim Moreau, Bertrand Lussier, Jean-Pierre Pelletier, Johanne Martel-Pelletier, Christian Bédard, Dominique Gauvin, Eric Troncy

**Affiliations:** ^1^Research Group in Animal Pharmacology of Quebec (GREPAQ), Faculty of Veterinary Medicine, Université de Montréal, C.P. 5000, Saint-Hyacinthe, QC, Canada J2S 7C6; ^2^Osteoarthritis Research Unit, Centre Hospitalier de l'Université de Montréal (CRCHUM), Notre-Dame Hospital, 1560 Sherbrooke Street East, Montreal, QC, Canada H2L 4M1; ^3^Department of Clinical Sciences, Faculty of Veterinary Medicine, Université de Montréal, C.P. 5000, Saint-Hyacinthe, QC, Canada J2S 7C6; ^4^Department of Pathology and Microbiology, Faculty of Veterinary Medicine, Université de Montréal, C.P. 5000, Saint-Hyacinthe, QC, Canada J2S 7C6

## Abstract

*Objective*. The aim of this randomized placebo-controlled trial was to evaluate the beneficial effect of a whole plant extract of *Brachystemma calycinum* D. Don (BCD) in naturally occurring osteoarthritis (OA) in dogs. *Methods*. Dogs had stifle/hip OA and poor limb loading based on the peak of the vertically oriented ground reaction force (PVF) measured using a force platform. At baseline, PVF and case-specific outcome measure of disability (CSOM) were recorded. Dogs (16 per group) were then assigned to receive BCD (200 mg/kg/day) or a placebo. The PVF was measured at week (W) 3 and W6. Locomotor activity was recorded throughout the study duration using collar-mounted accelerometer, and CSOM was assessed biweekly by the owner. *Results*. BCD-treated dogs had higher PVF at W3 and W6 when compared to Baseline (*P* < 0.001) and at W6 when compared to placebo-treated dogs (*P* = 0.040). Higher daily duration (*P* = 0.024) and intensity (*P* = 0.012) of locomotor activity were observed in BCD-treated dogs compared to baseline. No significant change was observed in either group for CSOM. *Conclusions*. Treatment with BCD improved the limb impairment and enhanced the locomotor activity in dogs afflicted by naturally-occurring OA. Those preclinical findings provide interesting and new information about the potential of BCD as an OA therapeutic.

## 1. Introduction

A group of experts recently emphasized the uses of companion animals suffering from naturally occurring diseases to accelerate the development of human therapeutics [1]. Hence to fulfill preclinical data, undertaking a trial in companion animals may represent an interesting way to provide additional evidence on the therapeutic potential of a new drug in development.

Naturally occurring osteoarthritis (OA) is common in companion animals, particularly in dogs. One study estimated that 20 per cent of dogs over one year of age are afflicted by the condition [2]. Traumatic insults to the cranial cruciate ligament (CCL) and hip dysplasia are among the arthropathies considered to be etiopathogenic of OA in this specie [3, 4]. Biological and biomechanical factors merge to induce and perpetuate OA, generating pain, lameness, and limb dysfunction [5, 6].

As for human, the therapeutics modalities to manage canine OA remains largely palliative, from which nonsteroidal anti-inflammatory drugs (NSAIDs) take place as the first line of treatment [7]. Only the alleviation of pain-related clinical sign(s) is claimed by actual modalities; structural benefits cannot be expected from current evidences and still represent a clinical challenge [8]. Therefore, there is an unmet need for OA therapeutics that combines disease modifying properties and the capacity to improve the locomotor disability. 

Surgical transection of the CCL in dogs impairs the limb function and creates lesions that mimic those encountered in human [9, 10]. In this model, the extract of *Brachystemma calycinum *D. Don (BCD), an indigenous plant of southwestern China (the Himalayas) has shown promising disease modifying potential against the development of OA lesions and limb impairment [11]. It was reported that the decrease in the levels of protease-activated receptor 2 (PAR 2), inducible nitric oxide synthase (iNOS), and matrix metalloprotease 13 (MMP-13) were the key factors tributary of the effect of BCD. However, whether BCD is effective against the locomotor impairment that prevails in dogs naturally afflicted with OA could be informative on its curative potential and need to be scrutinized.

With the idea of providing preclinical data to enhance the development of human therapeutics, the objective of this trial was to determine whether BCD can improve the locomotor disability seen in naturally occurring OA in dogs.

## 2. Materials and Methods

### 2.1. Design and Subject Selection

This study was a randomized, double-blind, parallel-group, placebo-controlled trial lasting 6 weeks. The trial was conducted under the approbation of the Institutional Animal Care and Use Committee (#Rech 1434) in accordance with the guidelines of the Canadian Council on Animal Care. All owners provided written informed consent.

Adult dogs weighed more than 20 kg and had radiographic evidence of OA exclusively at the hip or stifle joints. Radiographs (hips, stifles, and elbows) were obtained under sedation as described [12]. Hind limb lameness in association with the presence of OA was confirmed by a certified veterinary surgeon (B. Lussier). At the time of screening, all dogs were free of any compound purported to relief the clinical signs of OA according to washout periods ranging between 4 to 12 weeks. Hence, a 4-week washout period was respected for oral NSAIDs and a 6-week period for natural health products including fatty acid supplement, OA therapeutic diets, or treats. Dogs having received injectable pentosan polysulfate sodium or corticosteroid one year before the screening visit were not eligible. A 12-week period was requested for injectable polysulfated glycosaminoglycan, and hyaluronan, and for oral or topical corticosteroid. During the study, dogs were free of any type of medication except those prescribed for exo- and endoparasite control. Additional exclusion criteria were as follows: dogs with surgical repair of the CCL within 1 year prior to study initiation, dogs suffering from neurologic or other musculoskeletal lesions, dogs that underwent orthopaedic surgery within the past year, and dogs with CCL disease having gross instability (positive drawer motion upon orthopaedic exam).

### 2.2. Complete Blood Count and Biochemistry Panel

Each dog underwent routine blood hematology and biochemistry analyses (C. Bédard) in order to evaluate health status at study entrance and to ensure that physiologic disturbance did not occur following treatment administration (W6).

### 2.3. Randomization, Blinding, and Therapy Regimen

Thirty-two privately owned dogs were randomized. The restricted randomisation process was defined as a random permuted blocks randomisation, which included a block size of four, with two treatments (A and B) distributed in one-to-one ratio. In blocks of four, there are six possible block allocation sequences: (1) AABB; (2) ABAB; (3) ABBA; (4) BBAA; (5) BABA; (6) BAAB. The treatment allocation sequence was defined using a list of true random integers from 10 to 99 (http://www.random.org/). The block allocation sequence was defined using the first eight single digit of the true random numbers list, omitting numbers outside the range 1 to 6. Among the eight designated blocks of four, a true random integer from 1 to 8 served to define which block was excluded from the balanced attribution of locomotor activity recording. In each of the seven remaining blocks, a true random integer from 1 to 2 served to allocate motor activity recording to treatment A (i.e., when a 1 was generated, motor activity recording was allocated to the first treatment A for a given block). The same procedure was repeated to allocate locomotor activity recording to treatment B, leading to the randomized attribution of seven dogs in each treatment group for monitoring locomotor activity. The 32 treatment allocations (with or without locomotor activity recording) were transcript on individual cards in sequentially numbered, sealed, opaque envelopes to ensure concealment. The person responsible of the randomization process (D. Gauvin) and the treatment preparation was not involved in the enrolment and followup. The test agent or the placebo were blinded to treatment A or B by a third party (E. Troncy). At trial site, both treatments were labelled exclusively as treatment A or treatment B and were encapsulated identically. The trialists (B. Lussier, M. Moreau), the veterinary technicians, and all dog owners were blinded to which treatment (A or B) was given to each randomized subject. The key code revealing what referred to treatment A and B (BCD or placebo) was kept confidential by the third party and was revealed only after study completion and preliminary analyses.

The test agent (BCD extract) was obtained as previously described [11]. The test agent or the placebo (corn starch) was given at a dosage of 200 mg/kg/day (minimum 197 mg/kg/day, maximum 240 mg/kg/day) using a combination of capsules (Torpac Inc., NJ, USA) that contains between 1 to 5 g/capsule. Initially, some dogs (*n* = 14) received a 5-day placebo treatment to establish baseline values, particularly of locomotor activity (see 2.5 below). Treatments were given in the morning, before or after meal. Treatments were encapsulated in the same fashion. The dose of BCD was the one used previously in experimental CCL-sectioned dogs and was based on the dose administered in humans (120 mg/kg) according to the following formula: human dose  ×  [*k*_*m* human_/*k*_*m* animal_], where *k*_*m*_ is the surface area to weight ratio.

### 2.4. Force Platform Measurement

Peak of the vertically oriented ground reaction force (PVF) was measured at baseline (day 0), W3 (day 21), and W6 (day 42) at the trot gait (1.9–2.2 m/s) using a force platform, as previously described [12]. The PVF was reported and defined as the primary outcome of interest. Normalized PVF in percentage of body weight (% BW) from the first five valid trials was used for statistical purposes. To be eligible, dogs must have hind limb PVF value less than 66% BW which is consistent to minus one SD of the value measured in normal dogs [13]. When bilateral lameness was observed, the hind limb having the lowest PVF, in accordance with orthopaedic exam findings, determined which one was selected for evaluation, otherwise the dog was excluded. The change in PVF was the mean difference between a given week value versus baseline.

### 2.5. Locomotor Activity Recording

Accelerometer-based motor activity recording was done using Actical system (Bio-Lynx Scientific Equipment Inc., Canada) as described [14]. According to the balanced attribution of motor activity recording, collar-mounted accelerometers were worn by seven dogs per group for the entire treatment duration (42 days, 24 hour/day). In addition, collar-mounted accelerometers were worn during a short period (5 days) that preceded real treatment initiation. This period was used to establish baseline level of locomotor activity recording before treatment administration. During this period, the entire 14 dogs were attributed to receive a placebo treatment managed by the person responsible of the randomization process (D. Gauvin). The duration and intensity of motion were continuously monitored and expressed as counts every 2 minutes, giving 720 counts per day. Daily duration of active period (DDAP) referred to the time spent (expressed in hour) when the count exceeded 30 in term of intensity. This cut-off value was based on intern data and was used to discern active from inactive period [14, 15]. Daily averaged total intensity (DATI) referred to the mean of all counts per day (unitless). Among the 47 days of continuous recording, three periods of 120 hours were predefined: baseline (day −5 to day 0), first period (day 17 to day 21), and the second period (day 38 to day 42). Owners of dogs were requested to come at day −5 and back at day 0 to the investigation site to acquire other baseline data.

### 2.6. Case-Specific Outcome Measure of Disability

Assessment of at-home functional disability was done using CSOM as previously described [14]. Owners assessed the ability of their dogs to perform between two to five activities using a 5-point scale for each activity that ranged from no problem (0) to incapacity (4). Each activity was selected by the owner according to his/her own perception of what characterise(s) the disability of the dog. Assessments were done twice weekly using a specific form that was kept at home by the owner. For each dog, medians of the activity scores were determined at each assessment (13 assessments) and were then used for statistical purposes. Among the 13 assessments, three periods were predefined: baseline (1st assessment done at day 0), first period (day 3 to day 21), and the second period (day 24 to day 42).

### 2.7. Statistical Analysis

All statistical tests were two tailed with significance determined by reference to the 5% threshold. Equality of efficacy was the null hypothesis based on the primary endpoint (PVF). Pre trial log-transformed PVF data were analyzed with a repeated measures general linear mixed model that includes two fixed factors (time and group) and their interaction (time × group interaction), with trials and dogs nested in treatment group as random effects. The compound symmetry covariance structure was used for this analysis. *Post hoc* analyses were done with appropriate Bonferroni adjustments. Log-transformed DATI and DDAP were analyzed similarly to PVF (time (period) and group as fixed factors) and their interaction (time × group interaction) with days and dogs nested in treatment group as random effects. A repeated measures generalized linear model was used to analyze median CSOM data under poisson distribution function using independent working matrix. Fixed factors were time (period) and group and their interaction (time × group interaction) with assessments and dogs nested in treatment group as random effects. Scale factor was estimated by Pearson's chi-square. The last recording was carried forward in the event of missing data. Data are presented as mean (standard deviation).

### 2.8. Sample Size Calculation

According to previous works done in similar conditions [15], a sample size of 16 dogs/treatment group ensured that a difference of 4.2% BW in the primary endpoint (PVF) between BCD and control (placebo) dogs could be detected assuming 75% power, an SD of 4.5 and a 5% significance threshold.

## 3. Results

### 3.1. Animal Description

No clinically relevant changes were observed on physical examination, observation, haematological, and biochemical analyses in the entire study cohort. Baseline characteristics of the dogs stratified per group are presented in Table 1. Groups were well balanced according to the outcomes of interest as significant difference was not observed for the levels of PVF (*P* = 0.452), locomotor activity recording when expressed as DDAP (*P* = 0.751) and DATI (*P* = 0.869), and also for CSOM (*P* = 0.194).

### 3.2. Study Withdrawal

**Figure 1:**
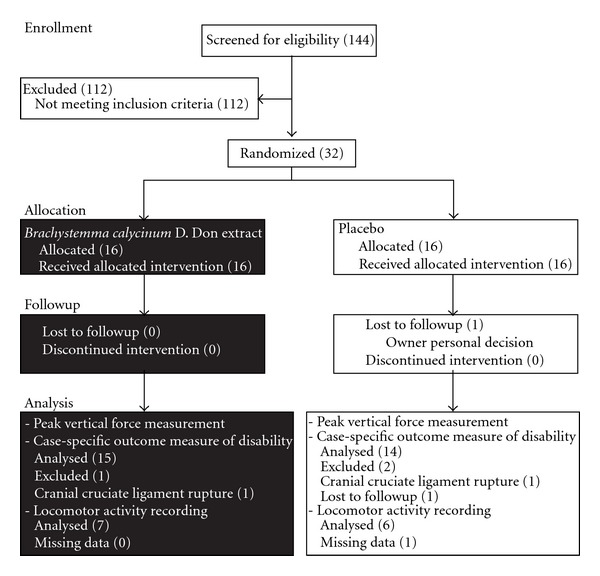
Flow chart of the study enrolment, randomization, followup, and analysis.

The numbers of dogs who were screened, randomly assigned, and analysed in each group are detailed in Figure 1. Last data (PVF and CSOM) were carried forward when incomplete data set were encountered.

### 3.3. Peak Vertical Force Measurement

**Figure 2:**
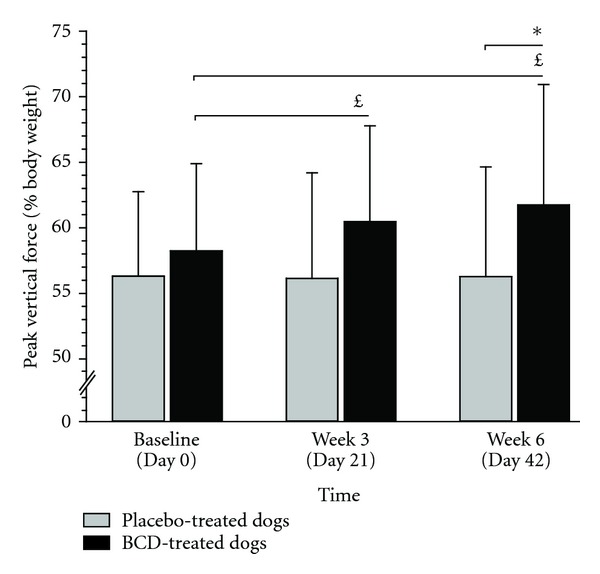
Mean (standard deviation) peak vertical force recorded in dogs having received either *Brachystemma calycinum *D. Don (BCD) or a placebo. Values are expressed as percentage of body weight. *Significantly different compared to placebo-treated dogs. ^*£*^Significantly different compared to baseline.

**Figure 3:**
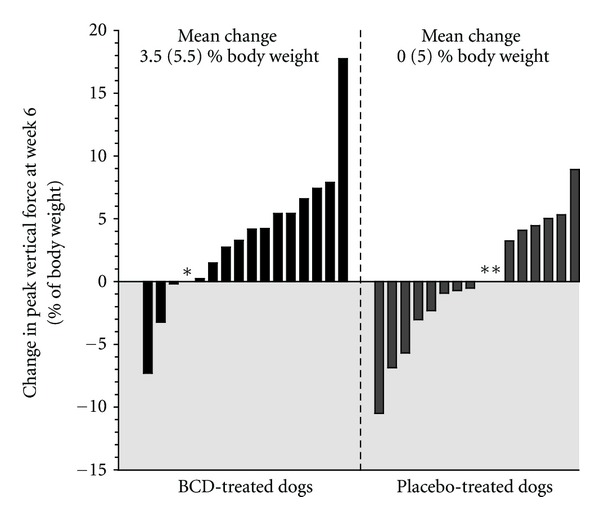
Individual changes in peak vertical force after 6 weeks of treatment with *Brachystemma calycinum *D. Don (BCD) or a placebo. Changes were the difference between Week 6 versus baseline. *Incomplete data were managed using last data carried forward method. Grey zone represent negative change (i.e., worsening).

The PVF (primary endpoint) generated by the disabled hind limb during the stance phase of the stride was increased in the overall study cohort (time effect; *P* < 0.001), without significant group effect (*P* = 0.129). Increment in PVF was mostly attributed to the changes observed in BCD-treated dogs. Hence, a significant time × group interaction (*P* < 0.001) was observed which means that groups evolved distinctively from baseline to the end of the study. More specifically, analyses revealed that the PVF of the BCD-treated dogs was significantly increased at W3 (*P* = 0.001) and at W6 (*P* < 0.001), when compared to baseline (Figure 2). At the opposite, neither W3 nor W6 value was significantly different than baseline in placebo-treated dogs. Analyses revealed that the change in PVF in BCD-treated dogs showed a tendency to be higher than placebo at W3 (*P* = 0.099), reaching significant level at W6 (*P* = 0.040).  Figure 3 presents the respective individual changes in PVF recorded at W6 as well as the mean change denoted in each group after 6 weeks of treatment.

### 3.4. Sensitivity Analyses

Sensitivity analyses were done using alternative forms of imputation to confirm the robustness of the results analysed with the last-observation-carried-forward method. Data management conducted with the exclusion of dog having incomplete data set provided an increase in PVF of 3.7 (5.6)% BW at W6 (*post hoc* comparison between groups at W6; *P* = 0.045). When positive data (+3.5% BW) were used to replace missing data, results were consistent with an increase in PVF of 3.7 (5.5)% BW at W6 (*post hoc* comparison between groups at W6; *P* = 0.040). When negative results (−3.5% BW) were used to replace missing data, results supported an increase in PVF of 3.3 (5.7)% BW at W6 (*post hoc* comparison between groups at W6; *P* = 0.043).

### 3.5. Locomotor Activity Recording

**Figure 4:**
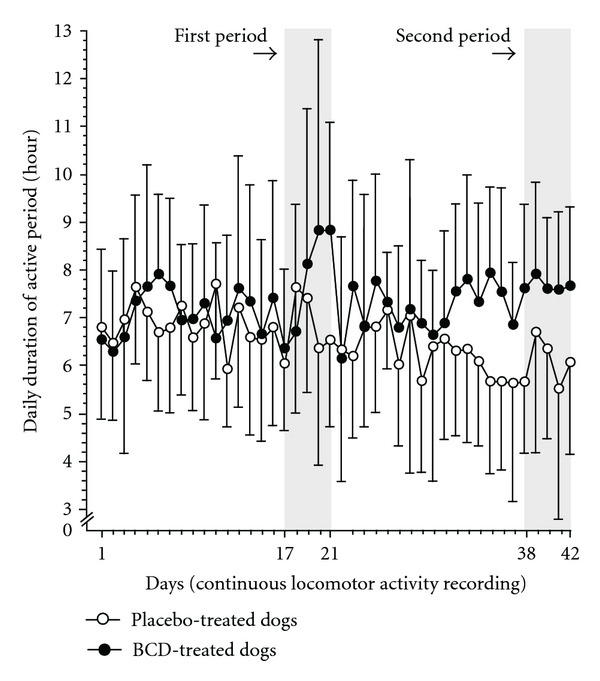
Temporal evolution of the locomotor activity recording over a 6-week period (42 days) in dogs receiving either treatment with *Brachystemma calycinum *D. Don (BCD) or a placebo. Data are the daily duration of active period and are expressed as mean (standard deviation). Periods were baseline (day −5 to day 0, not shown), first period (day 17 to day 21), and the second period (day 38 to day 42). At the second period, BCD-treated dogs had significantly higher daily duration of active period (*P* = 0.024) when compared to baseline.

The accelerometer recorded the motion of the dogs over the entire daily duration. The continuous recording was successful in 7 BCD- and 6 placebo-treated dogs. For DDAP, statistical findings were as follows: time effect (*P* = 0.032), group effect (*P* = 0.575), and time × group interaction (*P* < 0.001). Analyses revealed a tendency for higher DDAP in BCD-treated dogs during the first period (7.3 (1.7) h, *P* = 0.068), reaching a significant level at the second period (7.4 (1.3) h, *P* = 0.024) when compared to baseline (Table 1, Figure 4). Placebo-treated dogs had DDAP values at the first [6.8 (1.4) h] and the second period [6.2 (1.7) h] that did not differ from baseline (Table 1, Figure 4).

According to DATI, statistical findings were as follows: period effect (*P* = 0.103), group effect (*P* = 0.722), and period × group interaction (*P* = 0.006). In BCD-treated dogs, analyses revealed significantly higher DATI during the first period (233 (98), *P* = 0.042) and the second period (229 (85), *P* = 0.012) when compared to baseline (see Table 1). Placebo-treated dogs had DATI values at the first (199 (66)) and the second period (185 (75)) that did not differ from baseline (see Table 1). Neither DDAP nor DATI denoted any significant difference between groups at baseline, as well as during the first and second period.

### 3.6. Case-Specific Outcome Measure

**Figure 5:**
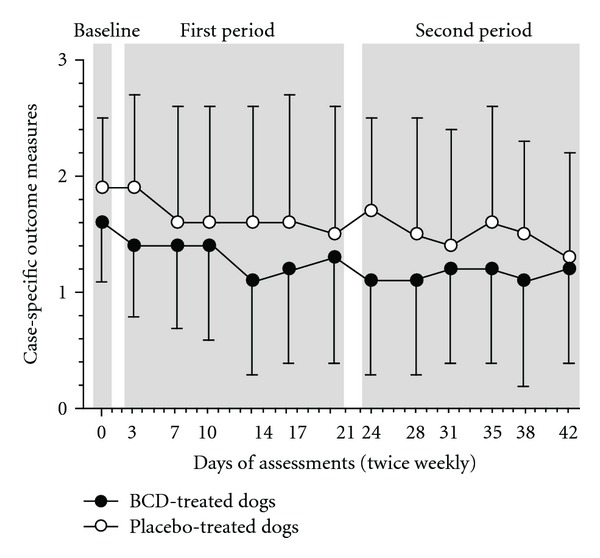
Temporal evolution of the case-specific outcome measures of disability (CSOM) over a 6-week period in dogs receiving either treatment with *Brachystemma calycinum *D. Don (BCD) or a placebo. Data are expressed as mean (standard deviation). Periods were baseline (day 0), first period (day 3 to day 21), and the second period (day 24 to day 42).

The CSOM assessed the severity of daily life disability in accordance with specific activities reported to be problematic, altered, and/or painful. Statistical findings were as follows: time effect (*P* = 0.003), group effect (*P* = 0.149), and time × group interaction, (*P* = 0.732).  Figure 5 presents the evolution of the CSOM recorded twice weekly over a 6-week period. Over time, no significant change in either group was observed. Placebo-treated dogs had mean CSOM of 1.6 (1.0) and 1.5 (0.9) at the first and second period, respectively. Mean values for BCD-treated dogs were 1.3 (0.8) and 1.1 (0.8) at the first and second period, respectively.

## 4. Discussion

In the experimental dog CCL model of OA, it was previously demonstrated that BCD treatment helps to reduce cartilage loss and improve functional disability [11]. According to those findings, hypothesis was then raised about the therapeutic potential of BCD in dogs naturally afflicted by the OA disease under curative conditions. Therefore, a randomized, double-blind, placebo-controlled trial was undertaken with the idea to fulfill preclinical evidences and to promote clinical trial in human OA. Based upon the measurement of the PVF defined as the primary endpoint, BCD improved the limb disuse in dogs afflicted by hind limb OA. When given once daily, improvements were seen as early as 3 weeks and reached further gain by a 6-week period. The magnitude of the therapeutic benefits was moderate in accordance with an effect size of 0.7 (95% confidence interval, 0.0–1.4).

Force platform is a recording instrument that measures the forces (such as vertical force) generated by the musculoskeletal system in close relationship with acceleration and mass of the body. Such platform has been considered as an objective measure of gait disability in OA patient [16–19]. In dogs with OA, pain-related limb disuse is discriminated by abnormally low PVF. An improvement is translated when an increment over initial condition occurs, as denoted following current therapies (Table 2). With respect to those clinical findings, the change in PVF provided by BCD (+3.7 (5.6)% BW at W6) was within the expected level of improvement provided by nonsteroidal anti-inflammatory drugs [20–22], COX-LOX inhibitor [15], complementary and alternative medicine [21, 23], and veterinary therapeutic diets [24–26]. The improvement demonstrated herein was translated into a willingness to load an average of ±1.4 kg on the painful afflicted limb.

The outcome measurements of the present study were in agreement with the pain, physical function, and patient global assessment included in the OMERACT-OARSI responder criteria [27]. There are actually no such criteria for OA clinical trials in dogs. Development of such an approach would be most useful to monitor the beneficial effects in a randomized clinical trial such as ours. In the absence of such consensus, an increment in PVF was instinctively considered as a positive response. According to Figure 3, 66% of the overall responders were BCD-treated dogs. At the opposite, 73% of dogs having worsened their condition probably due to natural fluctuations in disease severity (maturation effect) were placebo treated.

The monitoring of ambulatory activities using accelerometers is a reliable technique, providing continuous, unsupervised, objective monitoring of mobility [28]. In the field of OA, it is well known that afflicted patients suffer limitations in their walking ability as monitored using accelerometer [29–31]. In dogs, this device was deemed adequate for at-home activity monitoring [32] while being a valid tool to document the therapeutic outcome of an OA management [33]. 

In the present trial, we denoted that BCD-treated dogs had higher locomotor activity (intensity and duration) at the end of the treatment duration. While placebo dogs had similar intensity and duration of active period, BCD-treated dogs reached higher levels, gaining an hour of activity per day. Aerobic and strengthening exercises are beneficial in reducing pain caused by OA [34–36]. Therefore, the effect of BCD could have been translated into more active dog that rehabilitated the painful and disused limb toward better muscular strength, allowing animal to load more weight on the afflicted limb. Such association was previously demonstrated in OA dogs by our group [37]. Hence, higher levels of daily motion have been mirrored by an improvement in limb loading, which supports the benefits of physical rehabilitation [38].

The way both groups evolved according to the objective measures of function was not replicated by the assessment of daily life activity performance. Rather, the study cohort demonstrated an overall decrease in CSOM, without specific changes in the condition of BCD- and placebo-treated dogs. Longer treatment administration may preclude to a full monitoring of treatment efficacy for such level of PVF improvement, as previously denoted following 3 months of feeding a therapeutic diet in OA dogs [39]. Of note, CSOM was defined as complementary to PVF measurement, providing insights on different clinical aspects of the OA disease [14].

Whether BCD-treated dogs were improved through the preservation of joint structure, the relief of pain, or via a combination of these main aspects of the OA disease cannot be answered with regards to the present trial. However, from the previous study CCL model of OA [11], it was shown that action on key inflammatory mediators, such as iNOS and PAR 2, was tributary of the therapeutic potential of BCD. Under BCD treatment, lower levels of these mediators were encountered consistently with a protection against cartilaginous changes and better limb remission in CCL-deficient dogs.

Evidences indicate that PAR 2 participates in the development of experimental OA [40, 41]. Furthermore, PAR 2 activation has been shown to sensitize peripheral nociceptive receptors such as vanilloid, ATP-gated ion channels, and glutamate types [42–45]. Such sensitization contributes to the mechanical hypersensitivity and pain-related dysfunction that is pathognomonic of OA [46, 47]. When integrated, those findings support the putative therapeutic target of PAR 2 to limit the functional disability as well as the structural changes of OA.

A number of important limitations need to be considered: (1) the short duration of the study (6 weeks) for a chronic disease such as OA; (2) the absence of pharmacokinetic data on this plant extract may have precluded to suboptimal dosage; (3) the imputation method for missing data was the last-observation-carried-forward method. This approach preserved the sample size, assuming that the response remains constant at the last observed and that missing data were at random. Underestimation or overestimation of the treatment effect may have occurred. Noteworthy, data managed using different imputation methods provided similar results, supporting the robustness of the primary endpoint results.

## 5. Conclusion

This study provided clinical evidences of the beneficial effect of BCD extract through its improvement to the locomotor disability associated with naturally occurring OA in dogs. Using objective measure of spontaneous mechanical allodynia and discomfort, the daily administration of 200 mg/kg/day of BCD extract was efficient, enough to improve the limb disuse, and to enhance the locomotor activity. These preclinical findings hopefully may eventually prove to have relevance for the treatment of OA in man.

## Figures and Tables

**Table 1:** Baseline characteristics of the dogs stratified per group.

Characteristics	Groups	
	Placebo	*Brachystemma calycinum *D. Don
Age (year)	5.9 (1.9)	5.6 (2.5)
Sex (male/female)	6/10	8/8
Body weight (kg)	42.5 (7.5)	40.3 (11.4)
Peak vertical force (% BW)	56.3 (6.4)	58.2 (6.7)
Locomotor activity recording (over 5-day)		
Daily duration of active period (h)	6.7 (1.8)	6.5 (1.5)
Daily averaged total intensity (unitless)	191 (66)	194 (79)
Case-specific outcome measure of disability	1.9 (0.6)	1.6 (0.5)
Osteoarthritis-afflicted joint		
Hip (count)	10	11
Stifle (count)	13	12
Hip and Stifle (count)	7	7

**Table 2:** Selected studies that reported statistically significant changes (i.e., improvement) in peak vertical force following different therapeutic approaches in dogs naturally afflicted by osteoarthritis.

Therapeutic approaches	Authors	Changes in peak vertical force	Trial duration
		(% BW)	(sample size)
Nonsteroidal anti-inflammatory drugs			
Etodolac	Budsberg et al. [20]	2.3 (0.4)	8 days (34)
Carprofen	Moreau et al. [22]	2.4 [−3.4 to 17.0]	60 days (16)
Meloxicam	Moreau et al. [22]	4.7 [−4.9 to 92.2]	60 days (16)
Licofelone	Moreau et al. [15]	2.9 ± 1.7	28 days (13)
Carprofen	Hielm-Bjorkmanet al. [21]	3.2 [−8.2 to 11.8]	56 days (15)

Complementary and alternative medicine			
Elk velvet antler	Moreau et al. [23]	2.4 ± 0.7	60 days (25)
Multiherbal preparation	E. Troncy (internal data)	2.6 (2.1)	56 days (13)
Homeopathic preparation	Hielm-Bjorkmanet al. [21]	2.3 [−3.4 to 10.2]	56 days (14)

Veterinary therapeutic diets			
Omega-3 fatty acids	Roush et al. [26]	3.9 ± 1.3	90 days (22)
Green lipped mussel	Rialland et al. [24]	2.5 (4.2)	60 days (23)
Omega-3 fatty acids	Moreau et al. [25]	3.5 (6.8)	90 days (14)

% BW stands for percentage of body weight. Mean (standard deviation). Median (minimum to maximum). Mean ± standard error of the mean.
